# Ethanolic Neem (*Azadirachta indica*) Leaf Extract Prevents Growth of MCF-7 and HeLa Cells and Potentiates the Therapeutic Index of Cisplatin

**DOI:** 10.1155/2014/321754

**Published:** 2014-01-30

**Authors:** Chhavi Sharma, Andrea J. Vas, Payal Goala, Taher M. Gheewala, Tahir A. Rizvi, Arif Hussain

**Affiliations:** ^1^Department of Biotechnology, Manipal University, Dubai International Academic City, P.O. Box 345050, Dubai, UAE; ^2^Department of Microbiology and Immunology, Faculty of Medicine and Health Science, UAE University, P.O. Box 17666, Al Ain, UAE

## Abstract

The present study was designed to gain insight into the antiproliferative activity of ethanolic neem leaves extract (ENLE) alone or in combination with cisplatin by cell viability assay on human breast (MCF-7) and cervical (HeLa) cancer cells. Nuclear morphological examination and cell cycle analysis were performed to determine the mode of cell death. Further, to identify its molecular targets, the expression of genes involved in apoptosis, cell cycle progression, and drug metabolism was analyzed by RT-PCR. Treatment of MCF-7, HeLa, and normal cells with ENLE differentially suppressed the growth of cancer cells in a dose- and time-dependent manner through apoptosis. Additionally, lower dose combinations of ENLE with cisplatin resulted in synergistic growth inhibition of these cells compared to the individual drugs (combination index <1). ENLE significantly modulated the expression of bax, cyclin D1, and cytochrome P450 monooxygenases (CYP 1A1 and CYP 1A2) in a time-dependent manner in these cells. Conclusively, these results emphasize the chemopreventive ability of neem alone or in combination with chemotherapeutic treatment to reduce the cytotoxic effects on normal cells, while potentiating their efficacy at lower doses. Thus, neem may be a prospective therapeutic agent to combat gynecological cancers.

## 1. Introduction 

Therapeutic properties of neem (*Azadirachta indica*) have been recognized since ancient times and have been extensively used in ayurveda, unani, and homoeopathic medicine [1]. Many compounds such as limonoids, azadirone, azadirachtin, and flavonoids, having therapeutic potential, have been isolated from various parts of neem tree and have been evaluated for their pharmacological actions and plausible medicinal applications along with their safety evaluation. Recent studies have shown that neem possesses anti-inflammatory, antiarthritic, antipyretic, hypoglycemic, antigastric ulcer, antifungal, antibacterial, and antitumor activities [2–6].

The antineoplastic properties of neem are gaining attention due to its cancer preventive, tumor-suppressive, antiproliferative, apoptosis-inducing, antiangiogenic, and immunomodulatory effects via several molecular mechanisms [6, 7]. Neem or its derivatives have been shown to exert their antioxidant properties by decreasing TNF-*α*, increasing IFN-*γ*, and modulating antioxidant enzymes such as glutathione S-transferase (GST) and certain hepatic cytochrome P450-dependent monooxygenases [7–13]. It induces apoptosis via both the intrinsic and extrinsic pathways and induces cell cycle arrest via p53-dependent p21 accumulation and downregulation of the cell cycle regulatory proteins cyclin B, cyclin D1, p53, and proliferating cell nuclear antigen (PCNA) [11, 14–16]. Interestingly, when used in conjunction with chemotherapeutic drugs like cyclophosphamide, cisplatin, 5-fluorouracil, or with radiotherapy, it potentiates their antitumor effects by activating proapoptotic signaling and negating survival signaling along with attenuating their side effects [14, 17–20]. Notably, cisplatin, the first member of a class of platinum-containing anticancer drugs, is widely used for treatment of solid malignancies. Cisplatin has a number of side-effects that can limit its use: nephrotoxicity, nausea and vomiting, ototoxicity (hearing loss), electrolyte disturbance, and hemolytic anemia, etc. Also, the majority of cancer patients eventually develop cisplatin-resistant disease necessitating combination therapy approach using multiple chemotherapeutic agents or combining with chemopreventive agents [21, 22].

Based on the facts mentioned above, the present study aims to evaluate the chemopreventive potential of ethanolic neem leaves extract (ENLE) alone or concurrently with cisplatin on human breast (MCF-7) and cervical (HeLa) cancer cells, with the objective of studying its antiproliferative activity on cancer cells while decreasing the cytotoxic effects on normal cells. Also, the molecular targets of ENLE were delineated to elucidate its *in vitro* anticancer effects.

## 2. Material and Methods

### 2.1. Cell Culture

The human breast cancer cell line, MCF-7, and human cervical carcinoma cell line, HeLa were maintained in DMEM (Sigma, USA) supplemented with 10% fetal bovine serum (FBS) (Sigma, USA) and 100x Pen-strep (Sigma, USA) in a humidified atmosphere of 5% CO_2_ in air at 37°C. Lymphocytes were isolated from healthy non-smoking donors using HiSep Media (HiMedia, India) as per the manufacturer's instructions [23] and were maintained in RPMI media (Sigma, USA).

### 2.2. Preparation of Drug Solutions

5% ethanolic neem leaves extract (ENLE) was prepared as described previously by Subapriya and coworkers (2005) with slight modifications [24]. Briefly, 2.5 g of fresh mature neem leaves was ground to a fine paste in 50 mL of 100% ethanol and the slurry was air-dried in a shaking incubator at 37°C with intermittently stirring at 2 h and then left overnight. The powder obtained was weighed and resuspended in dimethyl sulphoxide (DMSO) (Sigma, USA) to prepare a stock solution of 80 mg/mL which was filtered through 0.2 *μ*m filter. Further dilutions were prepared in DMEM to require concentrations between 10 and 500 *μ*g/mL for treatment of MCF-7 cells, HeLa cells, and lymphocytes.

A stock solution of 3.3 mM of cisplatin (Cadila Pharmaceuticals Ltd., India) was used to make drug dilutions of varying concentrations (1–200 *μ*M) in complete medium.

### 2.3. Cell Viability Assay

The effect of ENLE and its combination with cisplatin, a chemotherapeutic agent, on the viability of MCF-7, HeLa, and lymphocytes was determined by 3-[4,5-dimethylthiazol-2-yl]-2,5-diphenyl tetrazoliumbromide (MTT) assay. The cells were plated in triplicate at a density of ~1 × 10^4^ cells/well in 200 *μ*L of complete culture medium containing 10–500 *μ*g/mL concentrations of ENLE alone for 48 and 72 h and 24 and 48 h for MCF-7 and HeLa, respectively, or a combination of ENLE (N1M and N2M = 50 and 100 *μ*g/mL; N1H and N2H = 10 and 50 *μ*g/mL) with cisplatin (C1M and C2M = 1 and 10 *μ*M in MCF-7; C1H and C2H = 1 and 5 *μ*M) for 48 and 24 h, respectively, for MCF-7 and HeLa in 96-well microtiter plates. After incubation for specified times at 37°C in a humidified incubator, MTT (5 mg/mL in PBS) was added to each well and incubated for 2 h. The absorbance was recorded on a microplate reader at the wavelength of 570 nm [23]. The effect of ENLE on growth inhibition was assessed as percent cell viability and was calculated as (OD of the drug-treated sample/OD of the nontreated sample) × 100, considering that the colorimetric signal is directly proportional to the number of viable cells. The EC_50_ (50% effective concentration) values were calculated from the dose-response curves.

### 2.4. Calculation of Combination Effects of Cisplatin and ENLE

Calculations of combination effects were expressed as a combination index (CI) as described previously [23]. CI analysis provides qualitative information on the nature of drug interaction, and CI, a numerical value, was calculated according to the following equation:

(1) $$\begin{array}{c} \text{CI}=\frac{{\text{C}}_{\text{A},x}}{{\text{IC}}_{x,\text{A}}}+\frac{{\text{C}}_{\text{B},x}}{{\text{IC}}_{x,\text{B}}}, \end{array}$$

where  C_A,*x*_ and C_B,*x*_ are, respectively, the concentrations of drugs A and B used in combination to achieve *x*% drug effect. IC_*x*,A_ and IC_*x*,B_ are the concentrations for single agents to achieve the same effect. A CI value <1, =1, or >1 represents, respectively, synergy, additivity, or antagonism of cisplatin and ENLE, respectively.

### 2.5. Detection of Apoptosis in MCF-7 and HeLa Cells after Treatment with ENLE

#### 2.5.1. Microscopic Examination

Morphological changes of MCF-7and HeLa cells were noted on treatment with ENLE at different concentrations (50, 200, and 500 *μ*g/mL) and time-points (48 and 72 h for MCF-7 and 24 and 48 h for HeLa) using normal inverted microscope (Labomed, USA) (Figures 2(a) and 2(b)). The untreated cells were used as negative control.

#### 2.5.2. Nuclear Morphological Studies

Apoptosis induction after treatment of MCF-7 and HeLa cells with ENLE at their respective EC_50_ concentrations and varying time-points (350 *μ*g/mL for 0, 48, and 72 h in MCF-7 and 175 *μ*g/mL for 0, 24, and 48 h in HeLa cells) was evaluated by the nuclear morphological changes associated with it using propidium iodide staining (Figures 3(a) and 3(b)) [22]. Briefly, ~10^6^ cells/mL cells were seeded on glass coverslips and incubated overnight in complete medium at 37°C. Further, cells were treated with ENLE at its EC_50_ for above mentioned time periods. At the end of the desired time interval, cells were fixed in a mixture of acetone: methanol (1 : 1) at −20°C for 10 min, washed with 1X PBS (pH 7.4) twice, and stained with propidium iodide (10 mg/mL in PBS) for 30 s in dark at RT. The coverslips were thoroughly washed with PBS and placed upturned onto a glass slide with mounting media (DPX). Slides were viewed at 515 nm under the Progress Fluorescent Microscope (Olympus, USA). The images were captured at 40x magnification.

#### 2.5.3. Quantification of Apoptotic Cells by Flow Cytometry

Cell cycle analysis of ENLE-treated MCF-7 and HeLa cells was performed by flow cytometry as described earlier (Figure 4) [23]. After treatment of synchronous cultures of MCF-7 and HeLa cells with ENLE at their respective EC_50_ concentrations at various time-points (350 *μ*g/mL for 0, 48, and 72 h in MCF-7 and 175 *μ*g/mL for 0, 24, and 48 h in HeLa cells), both adherent and floating cells were harvested, washed with phosphate buffered saline (PBS, pH 7.2), and fixed with ice-cold absolute ethanol at −20°C overnight. Cells were then washed with PBS prior to resuspending in a buffer containing PI (50 mg/mL), 0.1% sodium citrate, 0.1% Triton X-100, and 100 mg/mL of RNase A. The cells were analyzed using Flow cytometry (Beckman Coulter flow Cytometer FC500, CXP Version 2.2). The data was analyzed using the Beckman Coulter KALUZA 1.1 analysis software.

### 2.6. Expression Analysis of Various Genes Targeted by ENLE

Reverse transcription PCR was used to detect the expression of Bax, cyclin D1, CYP 1A1, and CYP 1A2 in response to treatment with ENLE at EC_50_ for varying time intervals (350 *μ*g/mL for 0, 48, and 72 h in MCF-7 and 175 *μ*g/mL for 0, 24, and 48 h in HeLa cells) (Figures 5(a) and 5(b)). Total RNA extraction from untreated and ENLE-treated MCF-7 and HeLa cells was carried out as per the manufacturer's instructions (GenElute Mammalian Genomic Total RNA Kit, Sigma, USA) at various time intervals. Further, total RNA was subjected to first strand synthesis as per manufacturer's protocol (ProtoScript M-MuLV Taq RT-PCR Kit, New England Biolabs, USA) followed by PCR using gene-specific primers [23, 25–28]. *β*-Actin was taken as an internal control. The PCR cycle was as follows: initial denaturation at 95°C for 5 min, followed by 35 amplification cycles (denaturation at 94°C for 30 s; annealing at 55°C for *β*-actin, CYP 1A1, and CYP 1A2, 56°C for Bax, and 54°C for cyclin D1; and extension at 72°C for 45 s), with final extension at 72°C for 7 min. Amplified products were visualized on a 2% agarose gel containing ethidium bromide.

## 3. Statistical Analysis

All data are expressed as means ± SD of at least 3 experiments. Fisher's exact test was adopted for statistical evaluation of the results. Significant differences were established at *P* < 0.05.

## 4. Results 

### 4.1. ENLE Shows Selective Cytotoxic Effects towards MCF-7 and HeLa Cells

**Figure 1:**
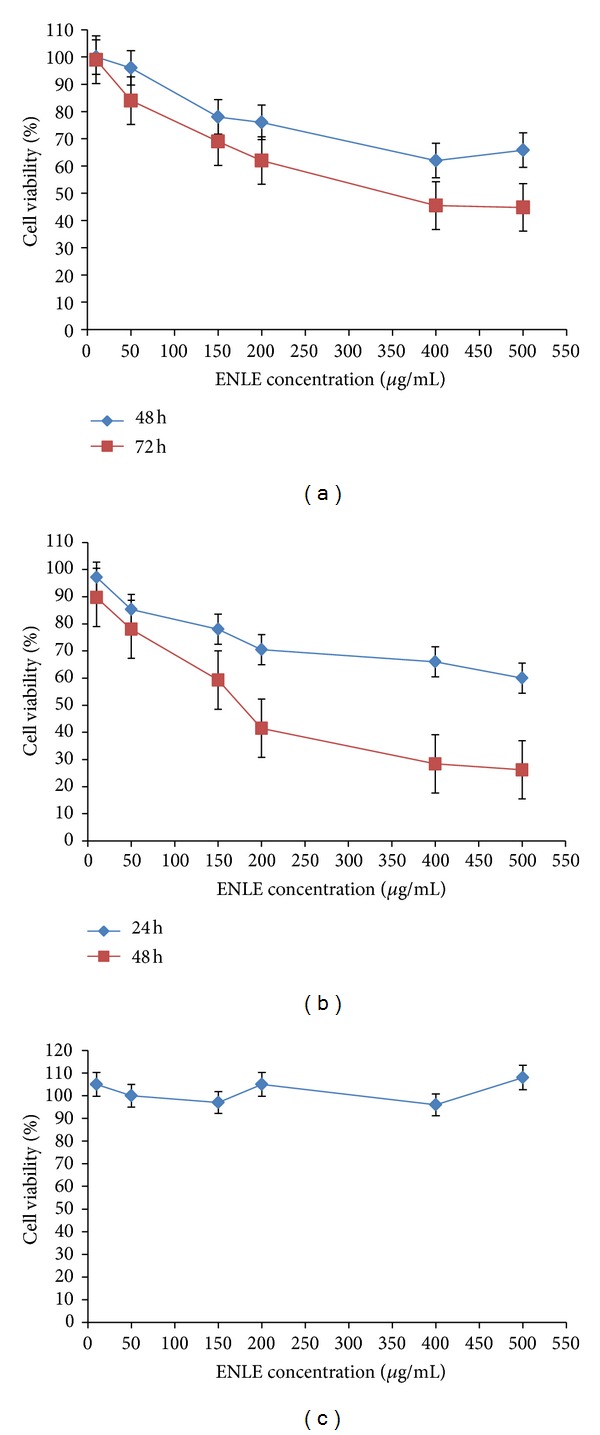
Differential cytotoxic effect of ENLE on MCF-7, HeLa, and lymphocytes. (a, b) MCF-7 and HeLa cells treated with ENLE at varying concentrations (10–500 *μ*g/mL), resulting in dose- and time-dependent growth inhibition. The EC_50_ for MCF-7 and HeLa cells was found to be 350 *μ*g/mL at 72 h and 175 *μ*g/mL at 48 h, respectively. However, lymphocytes did not show significant growth inhibition when treated with similar concentrations of ENLE for 24 h (c). Values are means + SD of three independent experiments. Each value with ENLE treatment differs from the control value (*P* < 0.05).

The antiproliferative effects of different concentrations of ENLE on MCF-7 cells, HeLa cells, and lymphocytes were evaluated by the MTT assay. MCF-7 and HeLa cells treated with increasing concentrations of ENLE ranging from 10 to 500 *μ*g/mL showed a dose- and time-dependent increase in cell death (Figures 1(a) and 1(b)). In MCF-7 cells, the EC_50_ was observed at 350 *μ*g/mL after 72 h treatment with ENLE, whereas in HeLa cells, it was found to be 175 *μ*g/mL in 48 h (Figures 1(a) and 1(b)).

Notably, to assess if ENLE possesses a safe cytotoxic profile, MTT assay was performed on lymphocytes isolated from a healthy nonsmoker adult at similar doses of ENLE (10–500 *μ*g/mL) (Figure 1(c)). No significant effect on cell viability was observed after treatment with ENLE for 24 h at these concentrations, thereby proving the fact that chemopreventive agents like neem can specially target the cancer cells (Figure 1(c)). This property of neem can be utilized for the purpose of cancer treatment because of its safety profile.

### 4.2. ENLE Induces Cell Death via Apoptosis in MCF-7 and HeLa Cells

#### 4.2.1. Morphological Changes Induced by ENLE on MCF-7 and HeLa Cells

**Figure 2:**
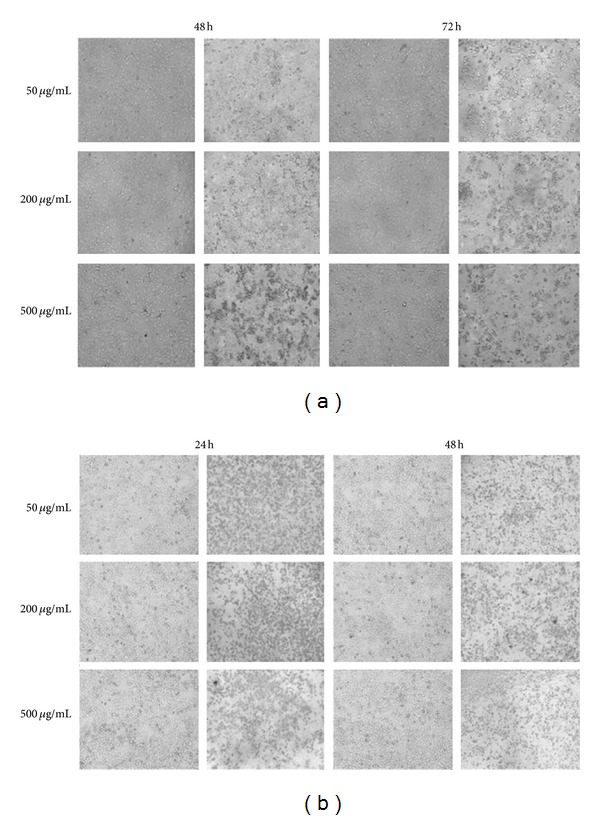
ENLE-induced morphological changes in MCF-7 (a) and HeLa cells (b) at varying concentrations and time-points. 50, 200, and 500 *μ*g/mL ENLE-treated (a) MCF-7 (for 48 and 72 h) and (b) HeLa cells (24 and 48 h) showed dose- and time-dependent increase in the morphological changes associated with cell death via apoptosis compared to the untreated cells (magnification 100x).

ENLE-treated MCF-7 (for 48 and 72 h) and HeLa (for 24 and 48 h) cells at the concentrations 50, 200, and 500 *μ*g/mL were observed under an inverted microscope and their morphological characteristics were noted. In comparison to untreated cells, ENLE-treated cells showed typical features of cell death at the morphological level such as rounding off of cells, cell shrinkage, and detachment from the substrate which accumulated in a dose- and time-dependent manner, thus indicating that ENLE induces cell death by apoptosis in these cells (Figures 2(a) and 2(b)).

#### 4.2.2. Nuclear Morphological Changes Induced by ENLE on MCF-7 and HeLa Cells

**Figure 3:**
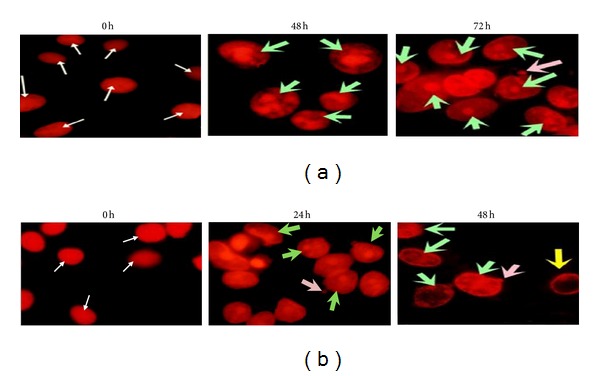
The nuclear morphological changes induced by ENLE treatment at various time intervals on (a) MCF-7 (for 0, 48, and 72 h) and (b) HeLa (for 0, 24, and 48 h) cells. Untreated MCF-7 and HeLa cells (0 h) showed large and prominent nuclei, indicating no significant characteristics of apoptosis (white arrows). On the other hand, ENLE treatment of these cells at their respective EC_50_ induced time-dependent increase in nuclear morphological changes characteristic of apoptotic cells such as nuclear condensation and fragmentation (green arrows), nuclear marginalization (yellow arrows), and appearance of apoptotic bodies (pink arrows) (magnification 400x).

ENLE-induced nuclear morphological changes characteristic of typical cell undergoing apoptosis were studied in MCF-7 and HeLa cells at their respective EC_50_ at various time-points. Untreated MCF-7 and HeLa cells appeared uniform in chromatin density with an intact nucleus. However, treatment of MCF-7 cells with ENLE for 48 and 72 h resulted in apoptosis-associated nuclear morphological changes like chromatin condensation and fragmentation along with appearance of apoptotic bodies (Figure 3(a)). Also, HeLa cells treated with ENLE showed similar changes in addition to chromatin marginalization (Figure 3(b)). With an increase in duration of ENLE exposure, there was a cumulative accrual of the said features consistent with apoptosis in both of the cell lines (Figures 3(a) and 3(b)).

#### 4.2.3. Effect of ENLE on the Cell Cycle Distribution

**Figure 4:**
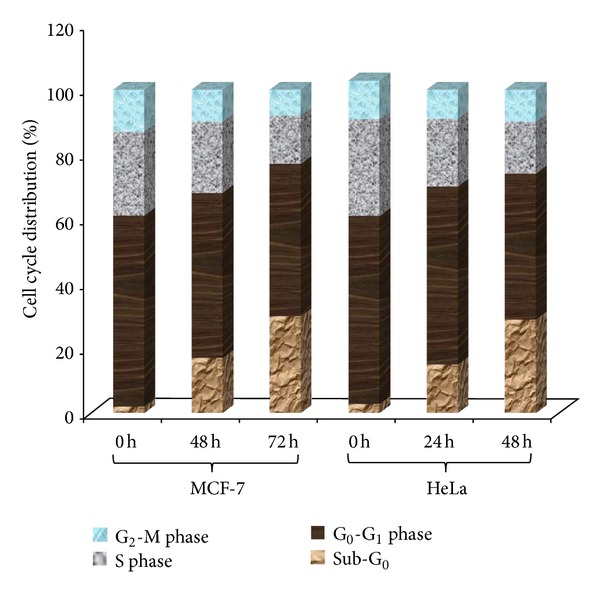
ENLE induces apoptosis in MCF-7 and HeLa cells as analyzed by flow cytometry. Untreated MCF-7 and HeLa cells showed normal distribution of cells in various phases of cell cycle, whereas when treated with ENLE at their respective EC_50_ doses, there was a significant increase in the number of cells in the sub-G_0_ phase of the cell cycle with increasing time of treatment (48 and 72 h for MCF-7 and 24 and 48 h for HeLa cells). The histogram shows % analysis of cells in the different phases of the cell cycle from a representative experiment (out of three individual experiments).

The effect of ENLE treatment on the cell cycle distribution of MCF-7 and HeLa cells was determined by flow cytometry after treatment of these cells with ENLE at their respective EC_50_ concentrations for 48 and 72 h for MCF-7 and, 24 and 48 h for HeLa cells. The untreated cells (0 h) showed appropriate distribution of cells in the different phases of cell cycle (Figure 4), while in the case of ENLE-treated cells, there was a significant time-dependent increase in the number of cells in the sub-G_0_ phase of the cell cycle (17 and 30% for MCF-7 after 48 and 72 h and 15 and 29% for HeLa after 24 and 48 h treatment) (Figure 4). This confirms that ENLE induces apoptotic cell death in these cells.

### 4.3. ENLE Treatment Significantly Modulates the Expression of Bax, Cyclin D1, CYP 1A1, and CYP 1A2

With the target of determining the effector genes involved in ENLE-mediated cellular responses in MCF-7 and HeLa cells, the expression of Bax, cyclin D1, CYP 1A1, and CYP 1A2 was analyzed before and after treatment with ENLE (48 and 72 h treatment for MCF-7 cells and 24 and 48 h treatment for HeLa cells). *β*-Actin was used as an internal control for comparison of samples.

**Figure 5:**
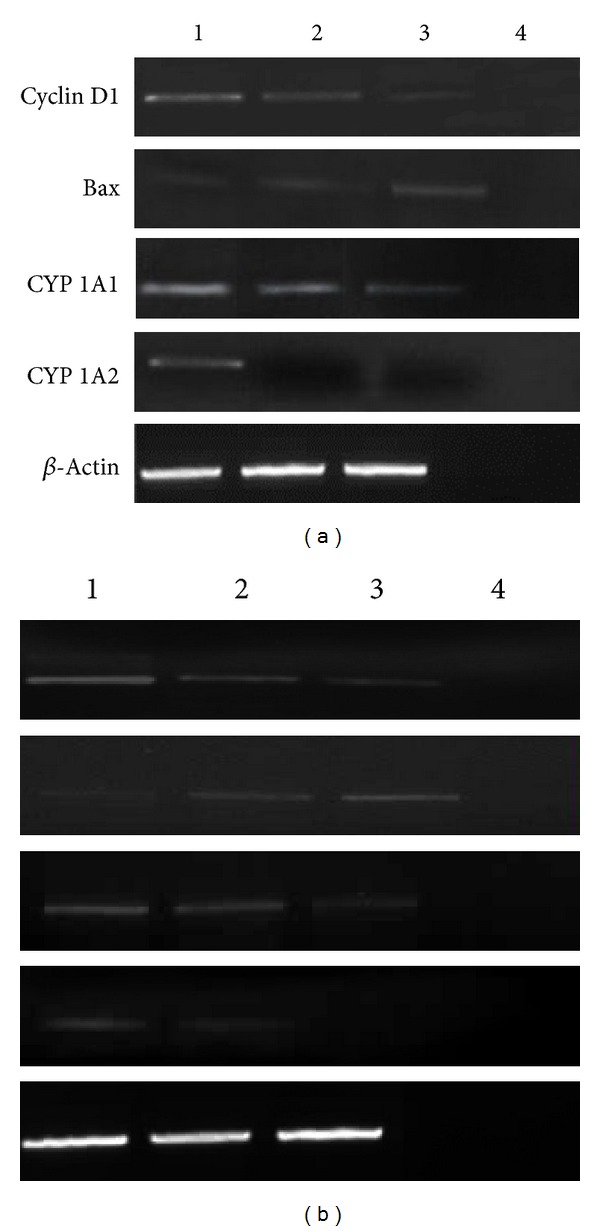
ENLE-treated MCF-7 (a) and HeLa (b) cells at their respective EC_50_ doses (48 h and 72 h for MCF-7 cells and, 24 and 48 h for HeLa cells) show a significant decrease in the expression of cyclin D1, CYP 1A1, and CYP 1A2 but a significant upregulation in the expression of bax in a time-dependent manner compared to untreated cells. *β*-Actin was used as an internal control. Lanes 1–4 represent untreated cells, cells treated with ENLE at their particular time of treatments, and negative control for RT-PCR, respectively.

The aberrant expression of cyclin D1, a key player in the progression of the cells from G1 to S phase, has been associated with the deregulated cell cycle control in many human cancers. It was found to be overexpressed in both the untreated MCF-7 and HeLa cells (Figures 5(a) and 5(b)). As shown in Figures 5(a) and 5(b), a significant, time-dependent inhibitory effect of ENLE was observed on the expression of cyclin D1 in both of the cell lines compared to untreated cells.

Bax, the first identified proapoptotic member of the Bcl-2 protein family, plays a major role in inducing apoptosis. In both untreated MCF-7 and HeLa cells, the expression of Bax was found to be low which significantly increased in ENLE-treated MCF-7 and HeLa cells in a time-dependent manner in comparison to the untreated cells (Figures 5(a) and 5(b)).

CYP 1A1 and CYP 1A2 are the members of the cytochrome P450 enzyme superfamily which act as drug metabolizing enzymes and lead to the accumulation reactive oxygen species forming ultimate carcinogens that are toxic to the cell and thereby leading to tumorigenesis. Expression of CYP 1A1 and CYP 1A2 was detected in untreated MCF-7 and HeLa cells (Figures 5(a) and 5(b)). However, in comparison to the untreated cells, ENLE treatment resulted in significant downregulation of these genes in both cancer cell lines (Figures 5(a) and 5(b)).

### 4.4. ENLE and Cisplatin Infusion Act Synergistically to Inhibit the Growth of MCF-7 and HeLa Cells

Since currently available chemotherapeutic drugs are associated with nonspecific cytotoxicity towards normal cells as well as development of chemoresistance, a combinational treatment with the natural dietary agents may serve as a better approach towards cancer treatment. In the present study, a combination of ENLE and cisplatin was evaluated to potentiate the chemotherapeutic index of cisplatin.

**Figure 6:**
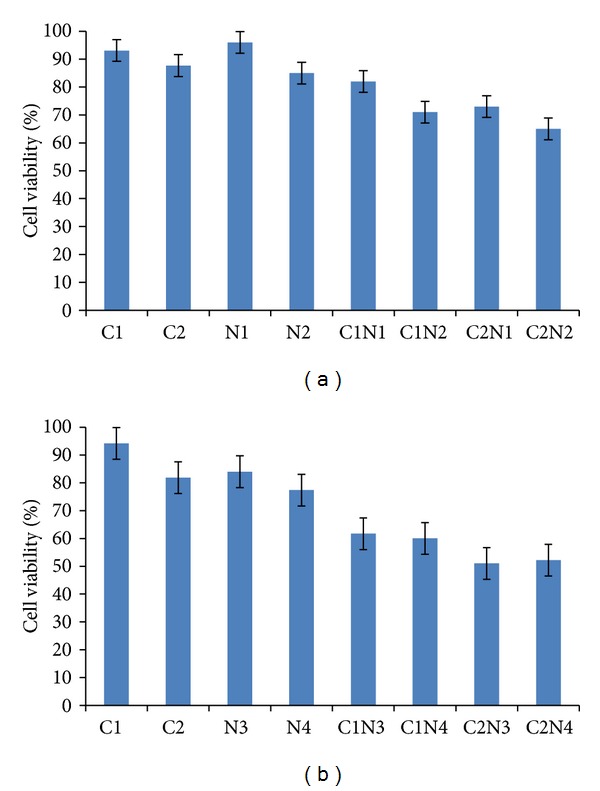
Simultaneous treatment of MCF-7 (a) and HeLa cells (b) with sublethal doses of cisplatin (C1 and C2) and ENLE (N1 and N2 for MCF-7 and N3 and N4 for HeLa) was found to induce synergistic decrease in viability of these cells (combination index (CI < 1)). Each value is a ratio of the level in the treated cells to that in the untreated control cells. Values are means ± SD of 3 independent experiments. Each value with cisplatin and ENLE treatment differs from the control value (*P* < 0.05).

The effect of concurrent treatment of MCF-7 and HeLa cells with different sub-lethal concentrations of cisplatin and ENLE for 48 and 24 h, respectively, was analyzed by cell viability assay. It was observed that 1 *μ*M of cisplatin (C1) used in combination with 50 (N1) and 100 *μ*g/mL (N2) ENLE resulted in a significant decrease in cell viability (82 and 71%, resp.) of MCF-7 cells as compared to either of the compounds alone (93.1% for C1 and 96 and 85% for N1 and N2) (Figure 6(a)). In HeLa cells, the combination of 1 *μ*M of cisplatin (C1) with 10 (N3) and 50 *μ*g/mL (N4) resulted in 61.7 and 60% (for C1N3 and C1N4) significant decrease in cell viability while individual drugs decreased the cell viability by 94.1% for C1 and 84 and 77.3% with N3 and N4, respectively (Figure 6(b)). Also, treatment of MCF-7 and HeLa cells with 5 *μ*M of cisplatin (C2) combined with N1 and N2 and N3 and N4, respectively, resulted in synergistic decrease in cell viability (73 and 65% for MCF-7; 51.0 and 52.2% for HeLa) as compared to individual doses (C2 = 87.7% and 81.8% for MCF-7 and HeLa) (Figures 6(a) and 6(b)). Combinational indices (CI) were calculated and CI were found to be less than 1 indicating a synergistic interaction between the two drugs at the doses used for both MCF-7 and HeLa cells.

## 5. Discussion

Regardless of recent advances in the prevention and detection of cancer and development of newer treatment modalities, cancer still as remains one of the most dreadful diseases due to the limitations of available treatment strategies [29, 30]. Research is under way to identify pharmacologically safe chemopreventive agents that can suppress the carcinogenesis process at various stages along with enhancing the therapeutic effects of conventional cancer therapy by tapping the potential of combinational approaches utilizing one or more synthetic or natural phytochemicals along with an effective drug such as chemotherapy [18, 23, 31, 32].

The present study focused on the antiproliferative properties of neem as a biosafe chemopreventive agent. It was found that treatment of MCF-7 and HeLa cells with ethanolic neem leaf extract (ENLE) inhibited the growth of these cells in a dose- and time-dependent manner (Figures 1(a) and 1(b)). The EC_50_ (effective concentration, the dose which reduces the viability of cells by 50%) of ENLE was found to be 350 *μ*g/mL on MCF-7 cells and 175 *μ*g/mL on HeLa cells after 72 and 48 h treatment, respectively. Notably, there was no significant effect of ENLE on the viability of lymphocytes pointing to its selective cytotoxicity towards the cancer cells and, thus, it provides a rationale for development of neem as a biosafe chemopreventive agent (Figure 1(c)). These results are in line with other studies which also showed that neem and its derivatives inhibit growth of various cancer cells such as prostate cancer, leukemia cells, head-and-neck squamous cell carcinoma cells, human choriocarcinoma cells, murine Ehrlich's carcinoma (EC), melanoma cells and exhibited only weak or no cytotoxic effect on normal cells [33–38].

Since carcinogenesis is associated with imbalances in the antiapoptotic and proapoptotic mechanisms leading to rapid and uncontrolled proliferation of cancer cells, therefore, inducing cell death is an important aspect in cancer prevention and therapy [39–41]. For this reason, the mode of cell death induced by ENLE in MCF-7 and HeLa cells was analyzed by changes in the cellular and nuclear morphology. MCF-7 and HeLa cells treated with various concentrations (50, 200, and 500 *μ*g/mL) of ENLE for 48 and 72 h and 24 and 48 h, respectively, were examined microscopically. ENLE-treated cells showed distinct features such as rounding off, cell shrinkage, and detachment from the matrix, which are the typical characteristics of cells undergoing programmed cell death (apoptosis) compared to untreated cells in which these morphological changes were absent (Figures 2(a) and 2(b)). Also, ENLE-treated MCF-7 and HeLa cells (at EC_50_ doses) showed discernible variations in the nuclear morphology of these cells, namely, formation of apoptotic bodies, nuclear condensation, fragmentation, and marginalization in comparison to uniform and intact nuclei of untreated cells (Figures 3(a) and 3(b)). These changes which are the hallmarks of apoptosis accumulated in the ENLE-treated cells in a time-dependent manner (Figures 3(a) and 3(b)).

Further, these results were verified by cell cycle analysis of MCF-7 and HeLa cells with or without ENLE treatment. Treatment of these cells at their respective EC_50_ concentrations for 48 and 72 h (MCF-7) and 24 and 48 h (HeLa) correspondingly resulted in increased proportion of cells in the sub-G_0_ phase of the cell cycle in a time-dependent manner compared to the untreated cells (Figure 4). These results conclusively prove that ENLE induces cell death in these cells mediated by the apoptotic pathway which are in agreement with previous studies that demonstrated apoptosis induction through various mechanisms such as inhibiting PI3 K/Akt pathway, decrease in Bcl-2/Bax ratio with increased expression of Apaf-1 and caspase-3, and cleavage of poly (ADP-ribose) polymerase was the mode of cell death induced by neem or its derivatives such as nimbolide in various cancers [6, 11, 14–16, 35–37, 42]. Thus, the activation of apoptosis is believed to be a critical therapeutic target for chemoprevention-based therapies.

ENLE-induced anticancer effects were then correlated with the modulation of gene expression of various effector molecules involved in cell cycle regulation, apoptosis, and drug metabolism. Cyclin D1, an important cell cycle regulator, is frequently overexpressed in several human cancers including breast and cervical [43, 44]. It was observed that untreated MCF-7 and HeLa cells showed a high expression of cyclin D1, which was significantly downregulated in a time-dependent manner in ENLE-treated cells (Figures 5(a) and 5(b)). These results are consistent with previous studies in which the antiproliferative action of neem and its bioactive components was associated with the downregulation of cyclin D1 expression in cancer cells [15, 16, 37].

Apoptosis is tightly regulated by a number of gene products that promote or block cell death at different stages. Bax, a proapoptotic gene, commits the cell to undergo programmed cell death in response to a wide range of cytotoxic stimuli [45]. The untreated MCF-7 and HeLa cells showed low expression of Bax (Figures 5(a) and 5(b)). However, on treatment of these cells with ENLE at their respective EC_50_ doses, there was a significant increase in the Bax gene expression in a time-dependent manner, accounting for the apoptosis-inducing activity of ENLE (Figures 5(a) and 5(b)). These results are in concordance with previous studies which found that neem and its component nimbolide upregulate Bax expression in human prostrate and colon cancer cells thus proving the potential of ENLE to induce apoptosis at the molecular level [36, 37, 46, 47].

CYP 1A1 and CYP 1A2, members of cytochrome P450 enzyme superfamily, are involved in the oxidative metabolism of endogenous compounds, such as steroids and fatty acids, and in the metabolism of foreign chemicals such as drugs, carcinogens, and other environmental pollutants. Increases in their expression have been linked to a higher risk of malignancies [48]. This is the first study in which the effect of ENLE on modulation of expression of CYP 1A1 and CYP 1A2 was analyzed. ENLE treatment resulted in significant decrease in their expression in a time-dependent manner as compared to untreated cells which showed relatively higher expression of these genes. This indicates neem can prevent or revert carcinogen induced accumulation of reactive oxygen metabolites which play a pivotal role in carcinogenesis [49]. Other studies have shown similar results in which quercetin, azadirachtin, and nimbolide exhibited free radical scavenging activity by downregulation of CYP 1A1 and 1A2 [50–54]. CYPs have also been correlated with bioactivation or inactivation of both carcinogens and anticancer drugs and thus modulation of their expression may be key determinants of cancer therapy [55, 56].

Conventional cancer treatments such as chemotherapy are associated with several cytotoxic effects; hence it was postulated that these drugs, when combined at lower dose with chemopreventive agents such as neem, can minimize the cytotoxicity while potentiating the therapeutic index [57]. It was found that cisplatin had nonspecific cytotoxicity towards both of the cancer cell lines (MCF-7 and HeLa cells) along with the normal cells (data not shown).

In context of the above mentioned facts, this is the first report analyzing the combined effect of ENLE and cisplatin on MCF-7 and HeLa cells. It was observed that sub-lethal doses of ENLE and cisplatin in various combinations (C1N1, C1N2, C2N1, and C2N2 for MCF-7 and C1N3, C1N4, C2N3 and C2N4 for HeLa cells) showed enhanced growth inhibitory effects in comparison to the individual doses as reflected in the CI less than 1 indicating a synergistic interaction between these drugs at the doses used (Figures 6(a) and 6(b)). Veeraraghavan and coworkers (2011) have also shown that neem induced radiosensitization radiotherapy [19]. Also, neem leaf preparation (NLP) has been shown to prevent the cyclophosphamide, cisplatin, and 5-fluorouracilinduced hematological complications [14, 18]. Therefore, combinations of chemopreventive agents with chemotherapeutic drugs may have immense prospects for development of therapeutic strategies to overcome chemotherapy associated resistance and side-effects in human cancers by synergistic crosstalk between two probable therapies.

## 6. Conclusion 

It can be inferred from the present study that neem alone or its infusion with cisplatin exhibits antineoplastic effects in breast and cervical cancers by inducing apoptosis and modulation of expression of effector molecules. Thereby, this study provides a rationale for extensive research and development work on neem for its better therapeutic utilization in cancer prevention and treatment.
